# A bipolar disorder patient becoming asymptomatic after adjunctive anti-filiarasis treatment: a case report

**DOI:** 10.1186/1471-244X-13-81

**Published:** 2013-03-13

**Authors:** Nora Hamdani, Raphaël Doukhan, Aline Picard, Ryad TAmouza, Marion Leboyer

**Affiliations:** 1 Faculté de médecine, Université Paris Est, Créteil 94000, France; 2 A. Chenevier, Pôle de psychiatrie, AP-HP, Hopital H. Mondor, Créteil 94000, France; 3 Equipe Psychiatrie Génétique, INSERM, U955, Créteil, 94000, France; 4 Fondation FondaMental, Créteil, 94000, France; 5 AP-HP, Laboratoire Jean Dausset d’Immunologie et d’Histocompatibilité & INSERM, UMRS 940, Hôpital Saint Louis, Paris, France; 6 Univ Paris Diderot, Paris, F75010, France

**Keywords:** Bipolar disorder, Filariasis, Antihelmintics, Cytokines, Mood stabilizers

## Abstract

**Background:**

Evidence suggests that neurotropic infectious agents might be involved in bipolar disorder. So far, few have been written for the association between parasitic infection and bipolar disorder. Filariasis is a parasitic disease acting ruthlessly via mosquitos and affecting more than 120 million people worldwide. We present here, to our knowledge, the first description of a filariasis infected manic bipolar disorder patient fully improved in terms of psychiatric symptoms by anti-heminthic treatment.

**Case presentation:**

The patient is a 31 years-old man native of Congo. At inclusion, he presented a severe manic episode with dangerous behaviour unresolved by classic treatments. A diagnosis of filariasis bancrofti infection was made after the discovery of a systemic hypereosinophilia. Therefore, a bi-therapy of anthelmintics was conducted allowing a successful improvement with clear reduction of agitation and aggressive behaviours that could not be attributed to a modification of psychotropic treatments or filarial encephalopathy or acute disseminated encephalomyelitis.

**Conclusion:**

The ineffectiveness of psychotropic treatment of a manic episode requires the evaluation of co-morbid medical conditions such as infections which can interfere with adequate mood stabilizing medication. Filariasis by inducing chronic inflammation and immunopathologic reactions seems to play a major role in infected affective disorders patients by changing levels of cytokines of the Th1 system or indirectly damaging the brain tissue. The beneficial combination of antihelmintics and mood stabilizers, in this case, could be explained by the potential of such association to downregulate neuroinflammation and excitotoxicity processes.

Altogether, these data pinpoint the requirement to explore the parasitic infectious status in case of bipolar disorder patients resistant to classic treatments and originating or living in endemic geographical areas.

## Background

Evidence suggests that neurotropic infectious agents might be involved in bipolar disorder. Therefore, specific treatments have been proposed as Amantadine, an antiviral compound against BDV (Borna Disease Virus), which seem to exert an antiviral and an antidepressive action in depressive infected bipolar type I patients [1]. Isoniazid which has been developed for prevention and treatment of tuberculosis, exhibits antidepressant properties. Minocycline, a tetracycline seems to reduce depressive symptoms among bipolar disorder patient [2] while clarithromycin, ciprofloxacin, erythromycin and amoxicillin [3,4] can lead to a manic episode and therefore are called “antibiomania” [5]. It has been suggested that mood stabilizing properties of macrolides could act via their interference in the mitochondrial functioning of neurons [6]. Filariasis is a parasitic disease acting ruthlessly *via* mosquitos in regions of Asia, Africa, Central, South America and Pacific Island nations, with more than 120 million people infected. 90% of this infection is caused by W. bancrofti with about 80 countries known to be endemic areas [7].The most important filarial disease for humans is lymphatic filariasis in which the adult worms are present in the lymphatic system [7]. Males are more frequently affected (sex ratio 10:1) in their third or 4^th^ decade [7]. The incubation period from mosquito bite may be comprised between 4 weeks and more than six months [7]. Usually asymptomatic, rare cases of neurological symptoms have been described. In monkeys, filarial worms were observed in the bulb, the protuberance, and the cervical cord [8]. In humans, several authors described cases with paralysis [9], hemiplegia [10,11], troubles of consciousness [12]. In addition, filariasis is suspected to cause insomnia, mental depression, irritability and severe headaches [13]. A relationship between central nervous symptoms and filariasis appears to be established in these cases, since the surgical removal of adult *L*.*boa*, and treatment of the *W*. *bancrofti* infections with specific treatment, was followed by the disappearance of symptoms. For to the authors, the neuropsychiatric symptoms observed may be due to the presence of adult worms in vital organs, but the possibility of systemic reactions caused by allergic sensitization of the host to filarial protein has to be considered, regardless of the location of the worms [13]. A post mortem study of filarial encephalitis showed a diffuse inflammation of the white matter (in a less extent, the grey matter), perivascular inflammation and a dramatic allergic reaction explaining serious neurologic symptoms [14].

Chemotherapy (antihelmintics) is now considered as the most cost-effective tool to potentially interrupt transmission even among asymptomatic patients.

We report successful improvement of manic symptoms when adding anthelmintics among an infected bipolar disorder patient refractory to usual treatments.

## Case presentation

The patient is a 31 years-old man with bipolar disorder (Diagnostic and Statistical Manual of Mental Disorders, DSM-IV criteria). The patient had a previous psychiatric history of depression and delusions 3 years before but the diagnosis of bipolar disorder was not evoked. It was the third relapse for the patient and two previous hospitalizations have been conducted in Congo. The patient was treated by haloperdidol before. There was no history of substance abuse. Native of Congo, he was admitted in a psychiatric ward for a manic episode with agitation, irritability, logorrhea, aggressive behaviour and delusions (persecutive, mystic and megalomaniac) (Young Mania Rating Scale, YMRS=43). The excitation and insomnia escalate to become dangerous, including physical violence needing physical restraint and seclusion. Pharmacological agents used to treat agitation including benzodiazepines, first- and second-generation antipsychotic drugs, and mood stabilizers showed no efficacy in spite of good compliance. The patient received clonazepam (8 mg) for several weeks with loxapine (400 mg) then cyamemazine (400 mg/day) and aripiprazole (25 mg) then olanzapine. Amisulpride was conducted at 1200 mg/ day for several weeks. Finally, valproic acid was administrated at 1250 mg/per day for several weeks. Neurological and somatic examination of the patient was normal. The MRI of brain was normal. Biological testing including routine blood counting and urine examination were normal with exception of a systemic hypereosinophilia which furhter lead to the diagnosis of a *filariasis bancrofti* infection using an immunodiagnosic method. However, we did not find any filariasis symptoms in our patient. A bi-therapy of anthelmintics including an association of ivermectin and albendazole was conducted. Two weeks after the initiation of anti-filariasis treatment, a successful improvement was observed with clear reduction of agitation and aggressive behaviours (YMRS=9) that could not be attributed to a modification of psychotropic treatments (modification of dosages), filarial encephalopathy or acute disseminated encephalomyelitis (absence of confusion, poor memory, incoordination, speech problems, lethargy, irritability, confusion ataxia, seizures and demyelinisation of the white matter). Euthymia was obtained simultaneously with the elimination of the parasite (according to immunodiagnosis status).

## Discussion

The ineffectiveness of psychotropic treatment of a manic episode requires the evaluation of co-morbid medical conditions such as infections, which can interfere with adequate mood stabilizing medication. The key pharmacological regimens in the management of lymphatic filariasis are diethylcarbamazine, albendazole, and ivermectin either used alone or in combination. However, combination therapy seems to have a better macrofilarial effect than monotherapy [15]. How could we explain the adjunctive effect of antihelmintics to mood stabilizers efficacy? To date, antiparasitic treatment has not been described to be effective among infected bipolar disorder patients. Ivermectin show unexpectedly poor penetration of the blood brain barrier [16]. One explanation could be given by the fact that ivermectin, which is a semi-synthetic macrolide, may have mood stabilizing properties via their interference in the mitochondrial functioning of neurons [6]. *A contrario*, albendazole seems to cross the blood-brain barrier and is prescribed to prevent filarial encephalopathy [17] or encephalomyelitis. It has been demonstrated that repeated albendazole treatment cause significant increases in production of the Th2 cytokines IL-5 and IL-13, by peripheral blood leukocytes [18]. According to the well known Th1 pathway overstimulation among manic patients with consequent proinflamatory cytokines production for review, [19], 2the Th2-mediated effect of albendazole can potentially lead to the inhibition of such Th1 pathway allowing anti-inflammatory responses and systemic inflammation downregulation. Hence, filariasis which induce chronic inflammation and immunopathologic reactions through pro-inflammatory Th1 cytokines (IFN-gamma, TNF-alpha, GM-CSF, IL-1alpha, and IL-8) [20] or immunopathological-related brain tissue damage could be alleviated by downregulation neuroinflammation and excitotoxicity properties of mood stabilizers in conjunction with antihelmintics.

## Conclusion

The treatment of bipolar disorder patients carrying a parasitic infection, by specific anti-infectious drugs could be useful thanks to their immuno-modulatory properties [21] and have to be evoked especially among patients native from or living in endemic countries.

## Patient consent

Written informed consent was obtained from the patient for publication of this case report and accompanying images. A copy of the written consent is available for review by the Editor-in-Chief of this journal.

## Competing interest

The authors declare that they have no competing interests.

## Authors’ contribution

NH supervised the inpatient treatment and hospitalization. NH suggested adding specific treatment after serological analysis. NH wrote the case presentation. RD and AP took in charge the patient and contribute to describe the case presentation. ML supervised the work of NH, AP and RD and ML & RT contributes to the redaction of the manuscript. All authors read and approved the final manuscript.

## Authors’ information

Ryad Tamouza and Marion Leboyer are co-authors.

## Pre-publication history

The pre-publication history for this paper can be accessed here:

http://www.biomedcentral.com/1471-244X/13/81/prepub
